# An Adult With Agenesis of Splenium of Corpus Callosum: A Case Report

**DOI:** 10.7759/cureus.26368

**Published:** 2022-06-27

**Authors:** Subhash Chander, Shahab Jazayeri, Julia Moulton, Shawnette Alston

**Affiliations:** 1 Internal Medicine, Saint Mary’s Hospital, Waterbury, USA; 2 Internal Medicine, Frank H. Netter MD School of Medicine, North Haven, USA

**Keywords:** syncope, epilepsy, agenesis of corpus callosum, heterotopic, seizure

## Abstract

A 22-year-old Hispanic immigrant presented to the emergency department after having a witnessed seizure. The patient was born and raised in Columbia and had a history of ventricular septal defect repair at the age of five years. Computer tomography (CT) of brain showed an unusual demonstration -“heterotopia of gray matter”- and the follow-up magnetic resonance imaging (MRI) revealed absence of splenium part of corpus callosum. The patient received a loading dose of IV antiepileptic medications and was then transitioned to oral dose. He was then discharged with seizure prophylaxis and referred for a follow-up at another tertiary care hospital for further workup. This case led to a management dilemma as the role of seizure prophylaxis in genetic brain malformations is not well established.

## Introduction

History of seizures is as old as human existence. Initially, seizures were considered a divine malady or a hellish possession and diagnosed as a condition stemming from the hand of sin and brought by the god of the moon [1]. The first seizure patient was a male from the Mesopotamia region and was described as neck turning, hand and feet tensing up, eyes staying wide open and frothing from mouth while person lay unconscious.

Agenesis of the corpus callosum is a developmental abnormality that can occur as partial or complete absence of carpus callosum. Majority of the time, it presents with an isolated finding; however, it can appear in concurrence with other development or congenital disorders. Males have a higher incidence of neurodevelopmental abnormalities than females, and most anomalies reported are due to cerebellar hypoplasia, short gut syndrome, congenital nephrosis, and frontonasal dysplasia; however, these patients also reported severe intellectual disability as well [2].

Different age groups also have different risk factors. Additional risk factors such as infections, tumors, head trauma, and metabolic derangements can occur at any age. However, cerebrovascular disease and end organ failure complications, which also increase the risk of seizure, are mostly associated with older age. Most adulthood cases present with underlying brain development malformation.

Among all developmental malformations, agenesis of the corpus callosum (ACC) remains unobtrusive developmental defect. Of the 30-45% of cases with ACC with identifiable genetic causes, 20-35% are caused by a mutation affecting a single gene [3]. Several causative gene mutations have been identified in ACC [4]. Autosomal dominant, autosomal recessive, and X-linked inheritance have been recognized in several syndromes associated with ACC [5,6]. Large number of human genetic syndrome is associated with the agenesis of the corpus callosum.

Patient with complete agenesis presents with developmental delays, speech problems, and difficulties with social interactions. Majority of these cases present early in their life. Minor defects remain unreported and sometimes notified in their second and third decade of life. Patient with early presentation of seizure also has higher chance of conversion to epilepsy after experiencing at least two seizures within 24 hours. Also, studies have reported three times higher risk of death in comparison to those without epilepsy [7].

## Case presentation

A 22-year-old Hispanic male who, at the age of five years, moved to the United States was brought to emergency department (ED) by the emergency medical services after having a seizure at work, witnessed by his coworkers. The patient works in an information technology management company, and while talking on the phone, he complained of weakness and an uneasy feeling in his extremities. The patient also endorsed feeling dizzy, being lightheaded, having blurry vision, and had aura before the seizure. His coworker who witnessed the event described the patient as very rigid followed by generalized jerking of his body for approximately 2 minutes before passing out. After waking up, he was confused for several minutes.

The patient is a healthy young adult, plays sports, and denies a previous history of seizures and substance use. He drinks alcohol occasionally; he last consumed it eight months prior to presenting in the ED. The patient’s past medical history was significant for ventricular septal defect which required surgical repair at the age of five years.

In ED, the patient was found to be hypertensive 143/48 mm Hg, tachycardic with heart rate of 111, and tachypneic. He was afebrile and maintained his O_2_ saturation on room air. The patient was awake, active, and alert. All physical examinations were negative for any pertinent findings. Emergent seizure precautions were taken, and a CT scan without contrast was obtained. The patient was given a loading dose of IV levetiracetam for seizure.

**Figure 1:**
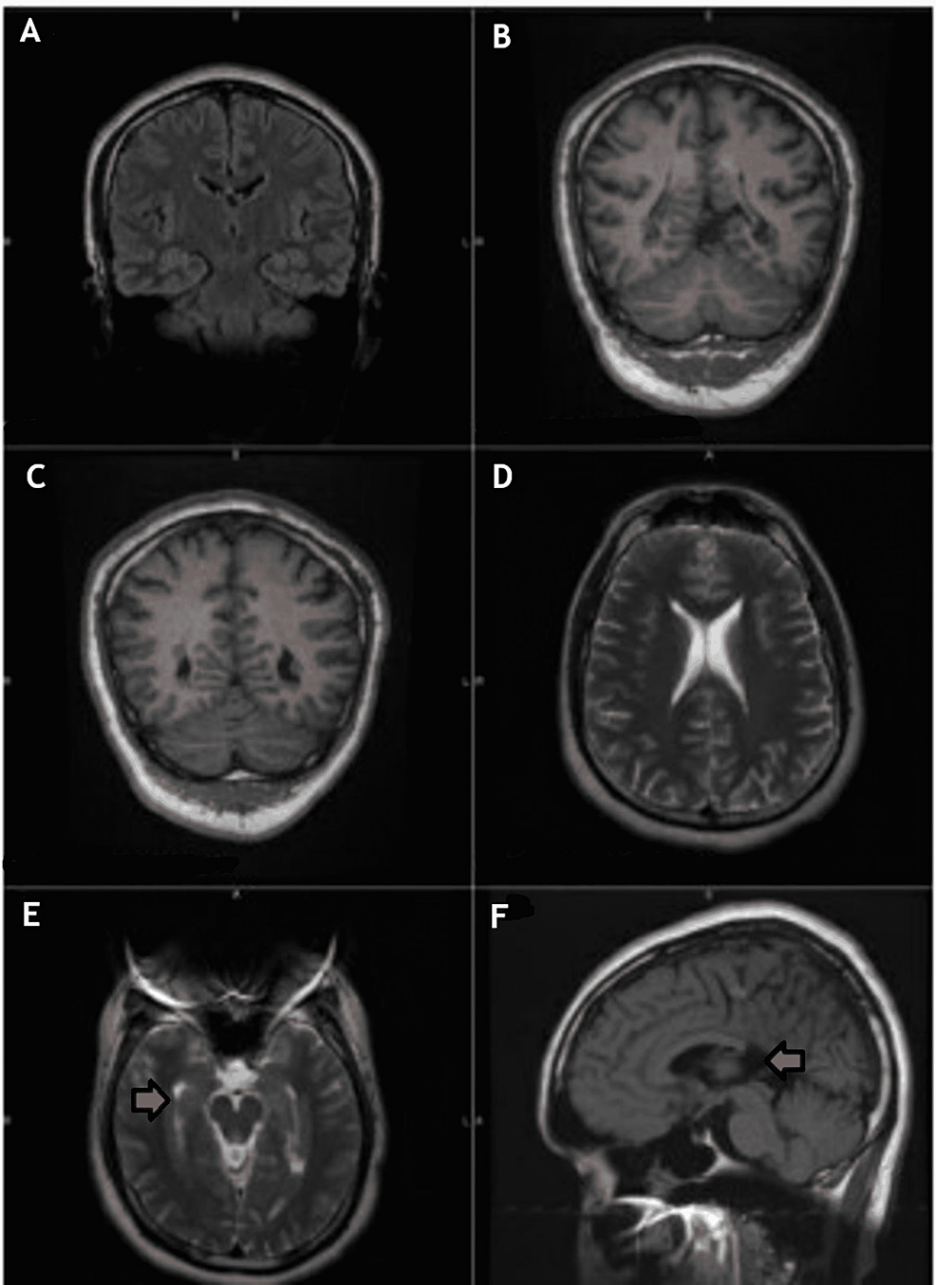
CT scan of the brain without contrast (A-F) Multiple foci of gray matter are seen along the subependymal and periventricular aspects of the temporal and occipital lobes. (E and F) The images of the brain showing development agenesis (arrows) of the splenium corpus callosum.

Laboratory investigation at the time of presentation is illustrated in Tables 1, 2. CT scan of the brain revealed a heterotopic gray matter lesion on the left ventricle. For further characterization, magnetic resonance imaging (MRI) was performed which showed heterotopic gray matter along the periventricular aspect of the temporal and occipital lobes bilaterally (Figures 1A-1F). Neurology was consulted and the patient was admitted to the telemetry floor.

**Table 1:** Laboratory investigations at the time of presentation

Test Name	Results	Normal Range
White blood cell count	6.2 × 10^9^/L	4.5-11 × 10^9^/L
Absolute neutrophil count	3.3 × 10^9^/L	1.8-7.7 × 10^9^/L
Hemoglobin	15.5 g/L	13.5-18.0 g/dL
Platelet count	290 k/μL	150-200 k/μL
Serum creatinine	0.9 mg/dL	0.7-1.2 mg/dL
Serum sodium	133 mmol/L	135-145 mmol/L
Serum potassium	3.6 mmol/L	3.5-5.1 mmol/L
Serum urea nitrogen	20 mg/dL	8-21 mg/dL
Estimated glomerular filtration rate	>60 mL/min/1.73	>60 mL/min/1.73
Serum bicarbonate	25 mmol/L	22-32 mmol/L
Serum total calcium	9.2 mg/mL	8.4-10.3 mg/mL
Serum ionized calcium	4.7 mg/mL	4.26-4.6 mg/mL
Aspartate aminotransferase	21 IU/L	13-36 IU/L
Alanine aminotransferase	28 IU/L	6-40 IU/L
Alkaline phosphatase	66 IU/L	45-115 IU/L
Total bilirubin	0.6 mg/dL	0-1.2 mg/dL
Magnesium	2.1 mg/dL	1.9-2.7 mg/dL
Phosphate	3.5	2.5-5.0 mg/dL
Blood cultures (2 sets)	No growth after 5 days	N/A

**Table 2:** Additional laboratory investigations (immunology/serology) TSH: thyroid-stimulating hormone; CK: creatine kinase; POC: point-of-care

Test Name	Results	Normal Ranges
Cortisol	13.3 ug/dL	6.7-22.6 ug/dL (AM) <10 ug/dL (PM)
TSH	0.46 ulU/mL	0.45-5.33 ulU/mL
CK	134	30-223 U/L
Lactic acid	1.0mmol/L	0.5-2.0 mmol/L
High sensitivity troponins	12	0-20 ng/L
Ethanol level	ND	ND
POC glucose	114	N/A
Creatinine	0.9	0.48-1.10
Urine drug screen		
Amphetamines	Negative	Negative
Barbiturates	Negative	Negative
Cocaine	Negative	Negative
Opiates	Negative	Negative
Oxycodone	Negative	Negative
Benzodiazepine	Negative	Negative
Phencyclidine	Negative	Negative
Cannabinoids	Negative	Negative

The patient did not have any seizures on the subsequent day. His diet was advanced, and antiseizure medication was continued. Follow-up EEG reported no seizure-like activity and no further workup was suggested by neurology. After 24 hours, the patient was discharged on oral levetiracetam prophylaxis and referred to a nearby tertiary care hospital for follow-up genetic testing. It was recommended to the patient to avoid driving for at least three months as per state law.

## Discussion

Seizure is the first clinical manifestation in up to two third of corpus callosum malformation. A patient with agenesis of the corpus callosum may have been admitted with other congenital abnormalities like ventricular septal defect, renal agenesis, and neurological disorders before the possibility of ACC is raised [7]. Many studies have reported higher risk of recurrent in children with congenital defects as compared to idiopathic causes and non-morphological defects [8,9].

Approximately 80% of the world’s people with epilepsy live in low to middle-income countries and most of the cases remain undiagnosed due to lake of healthcare resources, education, and parents' awareness [10]. Cultural beliefs, traditional practices, and social relationships play a vital role in daily life of majority of people in developing countries. More importantly, immigrants to western countries do not generally dissociate themselves from previously held ethnomedical beliefs about health, sickness, and care [11].

People with newly diagnosed seizure and neurological deficits present since birth including mental retardation or cerebral palsy reported the highest mortality. However, patients with epilepsy have up to threefold increase in mortality as compared to general population [12].

Genetic testing also helps guide the associated therapeutic options. In addition, genetic testing is often necessary for infants and young children with congenital defects with epilepsy syndrome. However, it should also be considered in older patients with a history suggesting an undiagnosed genetic epilepsy syndrome that began early in life. Recent studies have shown the effect of genetic mutation may cause both epilepsy and a cardiac conduction defect that gives rise to sudden death (Table 3) [13,14].

**Table 3:** Genetic syndromes with agenesis of the corpus callosum (ACC)

Syndrome	Chromosomal Region	Gene
Autosomal-dominant		
Apert syndrome	10q26	FGFR2
Basal cell nevus syndrome	9q22.3	PTCH
Miller-Dieker syndrome	17q13.3	LIS1
Rubinstein-Taybi syndrome	16p13.3	CREBBP
Autosomal recessive		
Andermann syndrome	15q13-q14	SLC12A6
Acrocallosal syndrome	7q13	GLI3
Joubert syndrome	6q23.2-q23.3	AHI1
Muscle-eye-brain disease	1q34-p33	POMGNT1
X-linked		
ATR-X syndrome	Xq13	ATRX
Craniofrontonasal syndrome	Xq12	EFNB1
Optiz G/BBB syndrome	Xq22	MID1
Proud syndrome	Xp22.13	ARX

Adults with single unprovoked first seizure and having prior brain insult and significant brain imaging abnormality have an increased risk of recurrence in the first two years [15]. Immediate anti-epileptic drugs (AEDs) therapy after first seizure may reduce the risk of second seizure in two years; however, it may not improve the quality of life. Longer treatment >3 years unlikely improves the prognosis for seizure remission [15-17].

## Conclusions

In our patient, the very first unprovoked seizure episode not only created a dilemma of living with this disease but also opened a wide discussion about the recurrence of seizures and future consequences of unknown other organ diseases. Better knowledge of his genetic makeup and treatment options will not only prevent future seizure episodes but also bridge the gap between favorable and unfavorable outcomes of the disease itself.
